# Rab32 promotes glioblastoma migration and invasion via regulation of ERK/Drp1-mediated mitochondrial fission

**DOI:** 10.1038/s41419-023-05721-3

**Published:** 2023-03-15

**Authors:** Pin Chen, Yanbing Lu, Binfeng He, Tao Xie, Chaolong Yan, Tengfei Liu, Silin Wu, Yuyang Yeh, Zeyang Li, Wei Huang, Xiaobiao Zhang

**Affiliations:** 1 Department of Neurosurgery, Zhongshan Hospital, Fudan University, Shanghai, China; 2 State Key Laboratory of Neuroscience, Institute of Neuroscience, Center for Excellence in Brain Science and Intelligence Technology, Chinese Academy of Sciences, Shanghai, China; 3 Department of Pulmonary and Critical Care Medicine, Zhongshan Hospital, Fudan University, Shanghai, China; 4 Cancer Center, Zhongshan Hospital, Fudan University, Shanghai, China; 5 Digital Medical Research Center, Fudan University, Shanghai, China

**Keywords:** Oncogenes, CNS cancer

## Abstract

The highly widespread and infiltrative nature of glioblastoma multiforme (GBM) makes complete surgical resection hard, causing high recurrence rate and poor patients’ prognosis. However, the mechanism underlying GBM migration and invasion is still unclear. In this study, we investigated the role of a Ras-related protein Rab32 on GBM and uncovered its underlying molecular and subcellular mechanisms that contributed to GBM aggressiveness. The correlation of Rab32 expression with patient prognosis and tumor grade was investigated by public dataset analysis and clinical specimen validation. The effect of Rab32 on migration and invasion of GBM had been evaluated using wound healing assay, cell invasion assay, as well as protein analysis upon Rab32 manipulations. Mitochondrial dynamics of cells upon Rab32 alterations were detected by immunofluorescence staining and western blotting. Both the subcutaneous and intracranial xenograft tumor model were utilized to evaluate the effect of Rab32 on GBM in vivo. The expression level of Rab32 is significantly elevated in the GBM, especially in the most malignant mesenchymal subtype, and is positively correlated with tumor pathological grade and poor prognosis. Knockdown of Rab32 attenuated the capability of GBM’s migration and invasion. It also suppressed the expression levels of invasion-related proteins (MMP2 and MMP9) as well as mesenchymal transition markers (N-cadherin, vimentin). Interestingly, Rab32 transported Drp1 to mitochondrial from the cytoplasm and modulated mitochondrial fission in an ERK_1/2_ signaling-dependent manner. Furthermore, silencing of Rab32 in vivo suppressed tumor malignancy via ERK/Drp1 axis. Rab32 regulates ERK_1/2_/Drp1-dependent mitochondrial fission and causes mesenchymal transition, promoting migration and invasion of GBM. It serves as a novel therapeutic target for GBM, especially for the most malignant mesenchymal subtype.

**Figure:**
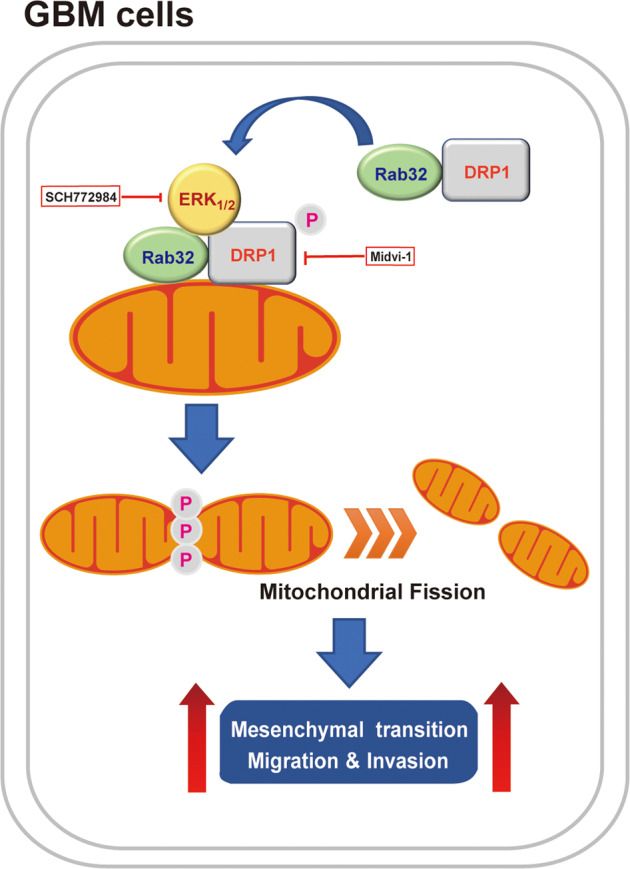
**Schematic of Rab32 promotes GBM aggressiveness via regulation of ERK/Drp1-mediated mitochondrial fission**. Rab32 transports Drp1 from the cytoplasm to the mitochondria and recruits ERK_1/2_ to activate the ser616 site of Drp1, which in turn mediates mitochondrial fission and promotes mesenchymal transition, migration and invasion of GBM.

**Schematic of Rab32 promotes GBM aggressiveness via regulation of ERK/Drp1-mediated mitochondrial fission**. Rab32 transports Drp1 from the cytoplasm to the mitochondria and recruits ERK_1/2_ to activate the ser616 site of Drp1, which in turn mediates mitochondrial fission and promotes mesenchymal transition, migration and invasion of GBM.

## Introduction

Malignant gliomas, which originate from glial cells and comprise anaplastic astrocytoma, anaplastic oligodendrogliomas, as well as glioblastoma multiforme (GBM), represent the most common intrinsic and highly aggressive primary brain tumor in the adults [1]. Among them, GBM belongs to the grade 4 astrocytoma according to the World Health Organization (WHO) Classification and is the most frequently occurring type, accounting for ~15% of all primary malignant brain tumors [2]. The current standard regimen includes maximal safe surgical resection followed by radiation and temozolomide (TMZ) chemotherapy, but the prognosis for GBM patients is still poor with median overall survival of merely 12–15 months and median progression-free survival (PFS) of ~6 months [3]. Previous studies revealed that the highly aggressive characteristic of GBM leads to infiltration of malignant cells into the surrounding normal brain tissue, makes complete surgical resection impossible, and results in poor prognosis with high recurrent rate [4, 5]. Unfortunately, the molecular mechanisms underlying GBM migration and invasion are not fully understood.

Recent genomic- and genetic-based molecular stratification were implemented to better understand the key factors of tumor malignant progression and divided the malignant glioma into four subgroups, including proneural, neural, classical, and mesenchymal phenotypes [6]. The mesenchymal phenotype exhibits a high expression of genes related to invasiveness, migration, and stemness, that leads to a poor clinical outcome [7]. Moreover, the proneural phenotype has a tendency to convert to the mesenchymal phenotype, analogous to the epithelial-mesenchymal transition (EMT) process that occurs in many cancers, which is an important driver of enhanced glioma invasion and malignant progression [8, 9]. But the potential causes of proneural-mesenchymal phenotype transition (PMT) remain unclear.

Mitochondria are highly dynamic organelles, which maintain their structural and functional integrity through two opposing processes known as fission and fusion. Switch between mitochondria-fission and -fusion is essential to maintain cellular physiological activity [10], while dysfunctional mitochondrial dynamics are closely linked to the development and progression of human malignant tumors, including lung, breast, and colon cancers [11–13]. Recent studies have shown that GBM also exhibits excessive mitochondrial fission and impaired mitochondrial fusion, which in turn promotes tumor malignancy and therapeutic resistance [14–17]. However, the mechanisms that trigger mitochondrial fission in GBM are not clear.

Rab32 belongs to the Ras-like small GTPase superfamily, which regulate membrane dynamics, including membrane fusion/fission, exocytosis, and cytoskeletal trafficking [18, 19]. A recent study showed that Rab32 serves as an oncogene in hepatocellular carcinoma with the potential to promote the growth and invasion of tumor cells, while a higher level of Rab32 was strongly related to the advanced tumor stage and poor survival in patients with hepatocellular carcinoma [20]. However, the role of Rab32 in GBM, as well as its underlying subcellular and molecular mechanisms remain unknown.

In the present study, we compared the expression level of Rab32 between normal brain tissues and different subgroups of gliomas based on both cancer public datasets and clinical tissue samples, and found elevated Rab32 expression in GBMs, especially in the most malignant mesenchymal subtype. Next, we tested the role of Rab32 on cultured GBM cells and uncovered that Rab32 was both essential and sufficient for mesenchymal transition, as well as the migration and invasion of GBM. We further revealed the cellular and molecular mechanisms of Rab32 that regulated Drp1-mediated mitochondrial dynamics in an ERK_1/2_ dependent manner. Finally, we integrated gene manipulation with malignant cell transplantation, and highlighted the importance of Rab32 in GBM formation and invasion in vivo.

## Materials and methods

### Antibodies and reagents

The antibodies were as follows: anti-Rab32 (Proteintech, #10999-1-AP, 1:1000), anti-MMP2 (Proteintech, #10373-2-AP, 1:1000), anti-MMP9 (Proteintech, #10375-2-AP, 1:1000), anti-β-Actin (CST, #4970, 1:1000), anti-N-cadherin (CST, #13116, 1:1000), anti-E-cadherin (CST, #3195, 1:800), anti-Vimentin (CST, #5741, 1:1000), anti-p-Drp1(Ser616) (CST, #3455, 1:1000), anti-p-Drp1(Ser637) (Abmart, #TD2980, 1:1000), anti-Drp1(CST, #8570, 1:1000), anti-Tom20 (Abcam, #ab56783, 1:1000), anti-p-ERK_1/2_ (CST, #4370, 1:2000), anti-ERK_1/2_ (CST, #4695, 1:2000). All secondary antibodies (HRP-conjugated anti-rabbit and anti-mouse, #A0208 and #A0216, 1:2000) were purchased from Beyotime Biotechnology (Shanghai, China). Anti-HA-tag (CST, #3724, 1:50 for IP and 1:1000 for western blot) and anti-IgG antibody (Servicebio, #GB23301, 1:100 for IP). Mdivi-1 (#S-7162), ERK_1/2_ inhibitor SCH772984 (#S-7101) were purchased from Selleck.

### Bioinformatic analyses

The gene expression profiles and clinical information of glioma patients were obtained from the Chinese Glioma Genome Atlas (CGGA, http://www.cgga.org.cn), the Cancer Genome Atlas (TCGA, http://cancergenome.nih.gov) and Repository for Molecular Brain Neoplasia Data (Rembrandt, http://caintegratorinfo.nci.nih.gov/rembrandt). These data had been analyzed through web-based tools GEPIA (http://gepia.cancer-pku.cn/) and Gliovis websites (http://gliovis.bioinfo.cnio.es/) [21].

### Human glioma tissue samples

Thirty-one glioma samples (WHO 2–4 grade) were collected during surgical resection from the Department of Neurosurgery, Zhongshan Hospital of Fudan University (Shanghai, China) between 2020 and 2022. Four non-tumor tissues were normal brain tissues obtained during corticectomy at the time of deep brain glioma exposure and resection, confirmed by postoperative pathology. Written informed consent for using clinical information and tissue samples was obtained from all patients. Our study was approved by the Ethics Committee of Zhongshan Hospital of Fudan University (Shanghai, China).

### Cell culture and gene transfection

Human GBM cell lines U87-MG (U87) and U251 were purchased from Cell Bank of the Chinese Academy of Sciences (Shanghai, China). Cells were cultured with Dulbecco’s modified Eagle’s medium (GIBCO, Waltham, MA, USA) supplemented with 10% fetal bovine serum (GIBCO, Waltham, MA, USA) at 37 °C in a humidified atmosphere of air containing with 5% CO_2_. The cell lines used for experimental studies were authenticated by STR profiling and tested for mycoplasma contamination.

Lentiviruses carrying Rab32 (OE-Rab32) or vectors, shRNA-Rab32 or shRNA-NC were purchased from Genomeditech Co., Ltd. (Shanghai, China). Stable U87 and U251 cells were established by lentiviral infection and puromycin selection as the manufacturer’s protocol and validated by western blot. Plasmids containing HA-Tag-Rab32 and empty vector were provided from Ph.D Binfeng He (Fudan University, Shanghai, China). These plasmids had been transfected respectively into cells using Lipofectamine 3000 Reagent (Invitrogen, CA, USA) for 48 h, and then the transfected efficiency was evaluated using western blot.

### Co-immunoprecipitation assay (Co-IP)

The interaction between Rab32 and ERK_1/2_ was analyzed by Co-IP using a Pierce Co-IP kit (Pierce, IL) according to the manufacturer’s instructions. Briefly, Anti-HA-tag antibody and anti-IgG antibody was incubated with Protein a Magnetic Beads for 3 h at 4 °C, respectively. Then, 1 mg of total protein was added to the mixture and incubated overnight at 4 °C in a refrigerator. The next day, the beads were washed four times with PBS containing 0.1% Tween-20 (PBST, pH 7.4) and collected. Then, samples were detected using western blot.

### Western blot analysis

Total protein was extracted from cells or tissues using RIPA cell lysis with protease inhibitor Cocktail and quantified by the BCA protein quantity kit (Beyotime, #P001). Mitochondrial protein extraction was performed using a mitochondrial isolation kit. The mitochondrial fraction was isolated using the Cell Mitochondrial Isolation Kit (Beyotime Biotechnology) according to the manufacturer’s instructions. The treated cells were first collected, 2.5 mL of mitochondrial isolation reagent was added, and then gently suspended for 15 min at 4 °C. The cell suspension was gently pulsed 10 times for 10 s each time with a homogenizer and continued to be centrifuged at 600 × *g* for 10 min at 4 °C, followed by 11,000 × *g* for 10 min at 4 °C. The residue was the mitochondrial fraction. Equivalent amounts of protein were separated by 7.5–12.5% SDS-PAGE and transferred to polyvinylidene difluoride membranes (0.45 μM PVDF, Millipore, USA). Then the membranes were blocked with skimmed milk for 60 min and incubated with the primary antibodies at 4 °C overnight. Next, the membranes were incubated with the corresponding HRP-conjugated secondary antibody for about 1–2 h at room temperature and the bands were visualized by ECL Western blotting substrate (Thermo Fisher Scientific, USA). The intensity of protein expression was measured using ImageJ software.

### Wound healing and transwell assay

Cell migration ability was measured by wound healing assay. Cells were seeded in a 6-well plate and cultured to reach 100% confluence, wounds were generated using a 200 μL micropipette tip. Cell cultured with serum free DMEM for further study. The images were acquired at 0 h and 24 h using an inverted microscope (Olympus BX51, Japan), and wound gap closure index were analyzed using ImageJ software.

Cell invasion assay was performed using a 24-well transwell chamber (8 μm pore size, #3422, Corning-Costar, USA) coated with Matrigel (#356234, BD Bioscience, USA). 2 × 10^4^ cells were suspended in 200 μL serum-free DMEM and seeded into a Matrigel pre-coated upper chamber. 600 μL DMEM with 20% FBS was added into the lower chamber. The non-invading cells in the upper chambers were removed gently with cotton tips after 24 h of incubation. The invading cells were fixed with 4% paraformaldehyde for 15 min and stained with 0.1% crystal violet for 20 min. These cells were counted in six randomly selected areas under an inverted microscope (Olympus BX51, Japan).

### Mitochondrial morphology analysis

Cells were seeded onto 35-mm confocal dishes, and grown to 30–50% fusion for immunofluorescence staining using a Tom20 antibody to observe mitochondrial morphology experiments. Then, mitochondrial network images were taken using a Nikon Eclipse Ti2 microscope with a 100 × 1.4 NA oil immersion objective, at a zoom 3 or 5, using Andor Zyla sCMOS camera and controlled by NIS-Elements software. Line averaging was set to 2 and signals were captured sequentially in two-channel mode.

### In vivo xenograft tumor models

All animal experiment procedures were approved by the Committee on Animal Research of Zhongshan Hospital, Fudan University (Shanghai, China) and were performed in accordance with the ethical principles and guidelines formulated by Shanghai Medical Experimental Animal Care Commission. Female athymic BALB/c nude mice (6–8 weeks old) were purchased from the Shanghai Experimental Animal Center of the Chinese Academy of Sciences and maintained under specific pathogen-free conditions for 1 week. The mice were randomly allocated to experimental groups prior to tumor cell implantation.

To established subcutaneous GBM model, U87 cells infected LV-shRNA-NC- or LV-shRNA-Rab32 (5 × 10^6^ cells in 100 µL PBS) were injected subcutaneously into the right back of nude mice (*n* = 5 for each group). Tumor size was measured every 5 days with a caliper regularly after tumor formation. The tumor volumes were calculated by the following equation: tumor size = length × (width)^2^/2. Five weeks after injection, the mice were euthanized and the tumors were harvested and further measured and weighed.

For the orthotopic animal model, mice were anesthetized and placed in a stereotactic frame (RWD Life Science, China), LV-shRNA-NC- or LV-shRNA-Rab32-infected U87 cells (5 × 10^5^ cells in 3uL PBS) were stereotactically injected into the right hemisphere of the mice’s brain (6 mice for each group) using the coordinates of the injection site at 1.5 mm anterior to the Bregma, 1.5 mm right of the midline, and 2 mm deep to the surface of the skull. Experimental animals were observed every two days until they showed moribund signs, then the mice were anesthetized and executed by rapid decapitation, tumors were extracted and preserved separately for IHC and western blot analysis.

### Statistical analysis

All experiment data were presented as mean values ± standard deviation (SD) from at least three independent experiments. Data analysis was performed by either Student’s t-test to compare between two groups or analysis of variance (ANOVA). *p* < 0.05 was considered statistically significant. All statistical analyses were performed using SPSS software, version 22.0 (Chicago, IL) or GraphPad Prism 8 software.

## Results

### Elevated Rab32 expression correlates with glioma malignancy and poor patient prognosis

**Fig. 1:**
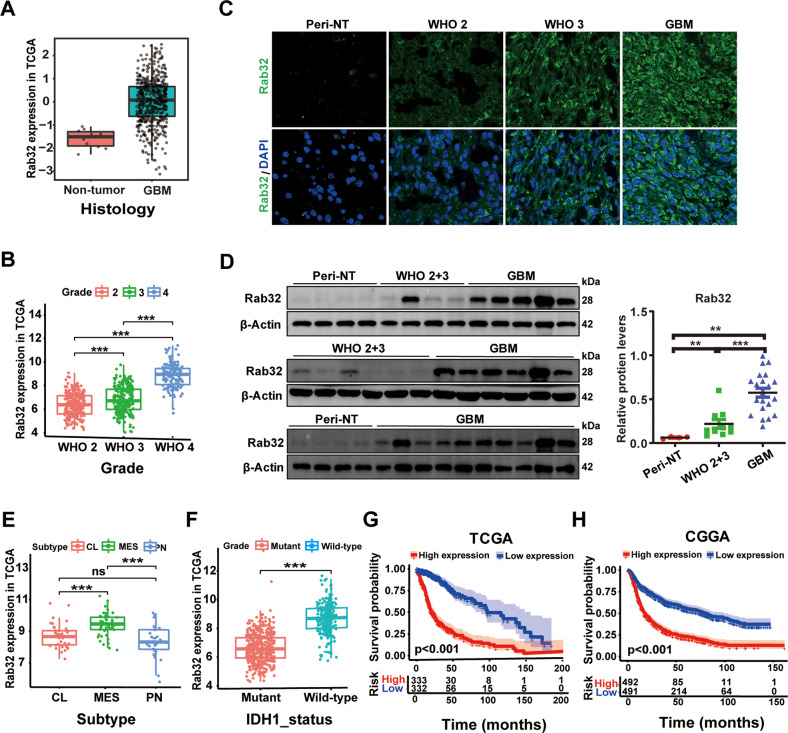
The increased expression of Rab32 indicates a poor prognosis of glioma malignancy. **A** Differential mRNA expression of Rab32 in GBM and non-tumor samples based on TCGA datasets. **B** The expression features of Rab32 in glioma of different clinicopathological grades in TCGA database. **C** Representative immunofluorescent staining of Rab32 expression in clinical glioma tissue and normal peritumor tissues. Scale bar = 50 μm. **D** Western blot analysis of Rab32 protein level in human glioma patient samples grade 2 (*n* = 4), grade 3 (*n* = 6), grade 4 (*n* = 21) and normal peritumor brain tissues (*n* = 4). β-Actin was used as a loading control. All data are shown as the mean ± SD (at least three independent experiments). *P < 0.05, **P < 0.01, ***P < 0.001. **E**, **F** The expression features of Rab32 in glioma of different molecular phenotype and IDH-status in TCGA database. **G**, **H** Kaplan–Meier analysis for correlation between Rab32 mRNA expression and survival of patients with gliomas in the TCGA and CGGA datasets.

In order to address whether Rab32 plays a role in GBM, we first analyzed the mRNA level of Rab32 in gliomas based on RNA-sequencing from TCGA, CGGA, and Rembrandt public datasets. Our analysis showed that Rab32 was more expressed in GBM compared to non-tumor samples (*p* < 0.05) (Fig. 1A and Fig. S1A). Furthermore, Rab32 expression levels were elevated significantly with the increase of WHO grade of glioma (*p* < 0.0001; Fig. 1B, Fig. S1B and C). We also evaluated the protein levels of Rab32 in a series of clinical specimens, which included 31 glioma tissues (21 cases of GBM and 10 cases of grade 2–3) and 4 normal peri-tumor tissues, by both immunohistochemistry and western blot analysis. In consistency with the above bio-informative analysis data, we found the protein level of Rab32 was much higher in GBM tissue than normal peri-tumor tissue, and positively correlated with tumor pathological grade (Fig. 1C, D).

We next studied the correlation between Rab32 expression and tumor molecular phenotypes based on the above public databases. The results showed that Rab32 was highly expressed in the mesenchymal subtype GBMs, but relatively lowly expressed in the proneural and classical subtypes (Fig. 1E, Fig. S1D and E). Since the isocitrate dehydrogenase 1 and 2 (IDH-1/2) mutation status is served as a critical biomarker for the guidance of the diagnosis and prognosis, and high-grade GBM with IDH1 mutations have a relatively better prognosis than IDH1 wild-type [22], we then checked the Rab32 protein level in both IDH1mutant and wild-type GBMs. Our data revealed that the expression of Rab32 was lower in the IDH-1mutant GBM than in the wild-type group (Fig. 1F and Fig. S1F). We further investigated the correlation between Rab32 expression and the survival of patients with glioma based on these databases. Kaplan–Meier plots demonstrated that the high level of Rab32 expression was correlated with poor overall survival of glioma patients (Fig. 1G, H and Fig. S1G). These findings suggested that high expression of Rab32 led to the poor prognosis of glioma patients, especially in the mesenchymal subtypes, the most migratory and invasive GBM.

### Rab32 regulates migration, and invasion of GBM cells

**Fig. 2:**
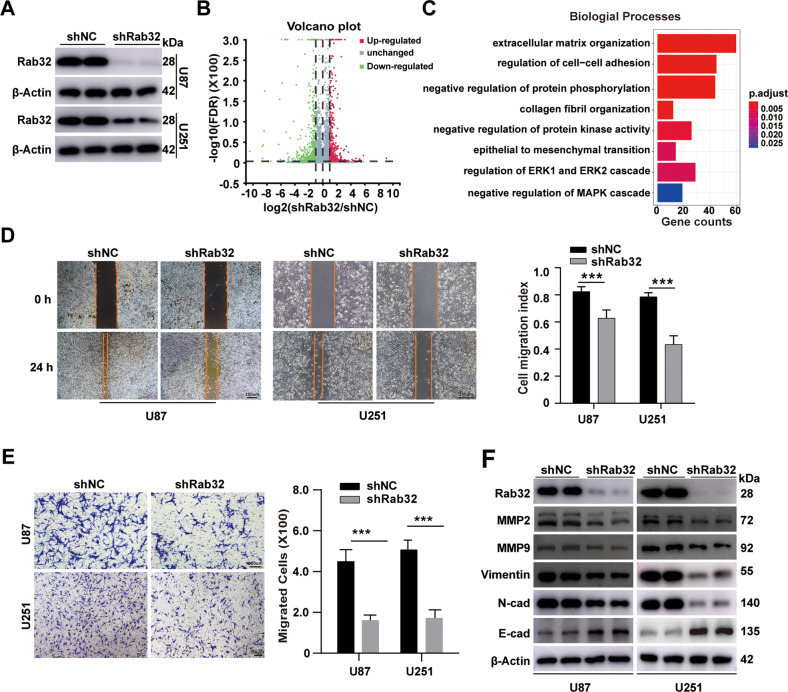
Rab32 knock-down inhibits cell migration, invasion, and mesenchymal transition of glioma. **A** Western blot analysis of the expression of Rab32 after U87 and U251cells were infected with LV-Rab32 shRNA. β-Actin was used as a loading control. **B** Gene expression profiling was performed in Rab32 knockdown U87 cells. Enrichment analysis of differentially expressed genes (DEGs) was presented as a volcano plot; genes with more than twofold significant changes were selected. **C** GO analyses were conducted to predict the potential molecular functions of Rab32. The size of the dots represents the number of genes enriched in each analysis. **D** A wound-healing assay was employed to analyze the migratory ability of Rab32-silenced U87 and U251 cells. Scale bar = 200 μm. **E** The invasive capacity of Rab32-silenced U87 and U251 cells was analyzed using a transwell invasion assay. Representative images of cells crossing the membrane are shown. Scale bar = 100 μm. **F** Western blotting analysis was performed to detect the MMP2, MMP9, Vimentin, N-cadherin, and E-cadherin expression levels. β-Actin was used as a loading control. The data are shown as the mean ± SD of three replicates. ***P < 0.001 vs. NC groups.

To determine the potential role of Rab32 in the aggressiveness of glioma, we evaluated the effect of Rab32 in the migration and invasion of GBM cells, U87 and U251. Firstly, Rab32 was silenced using Rab32-shRNA (Fig. 2A), and the differential gene expression profile between Rab32-knockdown and control cells was detected using RNA-sequencing. Silence of Rab32 upregulated a total of 460 genes, while downregulated 505 genes (fold change > 2; false discovery rate ≤ 0.001) (Fig. 2B). Gene ontology (GO) indicated that several important terms relating to cell mesenchymal transition, invasion and migration process were enriched. These included the extracellular matrix organization, regulation of cell–cell adhesion, collagen fibril organization, as well as epithelial to mesenchymal transition (Fig. 2C).

We further verified the effect of Rab32 knockdown on the migration and invasion capacity by wound healing assays and cell invasion assays, which measure the scratch-healing stripes and penetrated cells, respectively. Our results showed that Rab32 knockdown significantly reduced the migratory capacity (Fig. 2D) and suppressed the invasion ability of U87 and U251 cells (Fig. 2E).

Excessive migration and invasion are characteristic of malignant brain tumors, and matrix metalloproteinases (MMPs), especially MMP2 and MMP9, play an essential role in the invasion of GBM cells [23]. The occurrence of epithelial-mesenchymal transition is recognized as an important contributor to the enhanced acquired migratory and invasive capacity of GBM tumor cells and is notably characterized by the upregulation of mesenchymal markers such as N-cadherin and Vimentin, as well as the decreases of epithelial marker E-cadherin [24]. We then validated the effect of Rab32 on the aggressiveness of GBM cells at the molecular level. The results showed that Rab32-knockdown reduced the expression of invasive-related and mesenchymal-related proteins MMP2, and MMP9, Vimentin, and N-cadherin, whereas elevated the protein level of epithelial marker E-cadherin in U87 and U251 cells, indicating the necessity of Rab32 in proneural to mesenchymal transition (Fig. 2F, Fig. S2A and B). In contrast, when we over-expressed Rab32 in U87 and U251 cells, data from our wound healing assay, cell invasion assay, and protein analysis together showed that Rab32 was sufficient to enhance the migration, invasion, and mesenchymal transition (Fig. S2C–H).

In addition, we evaluated the effect of Rab32 on glioma cell proliferation and apoptosis by clone formation assay as well as the Propidium Iodide-Annexin V assay. The results of the clone formation assay showed that the shRab32 group formed fewer clones than that of the shNC group in both U87 and U251 cells. However, the apoptotic rate in the shRab32 group is no different from that of the shNC group. In contrast, overexpression of Rab32 increased clonal size and number without affecting apoptosis. (Fig. S3A–B).

These results collectively demonstrated that Rab32 promoted the proneural to mesenchymal transition, as well as enhanced the proliferation, migratory and invasive capabilities of GBM cells.

### Rab32 promotes Drp1-mediated mitochondrial dynamics

**Fig. 3:**
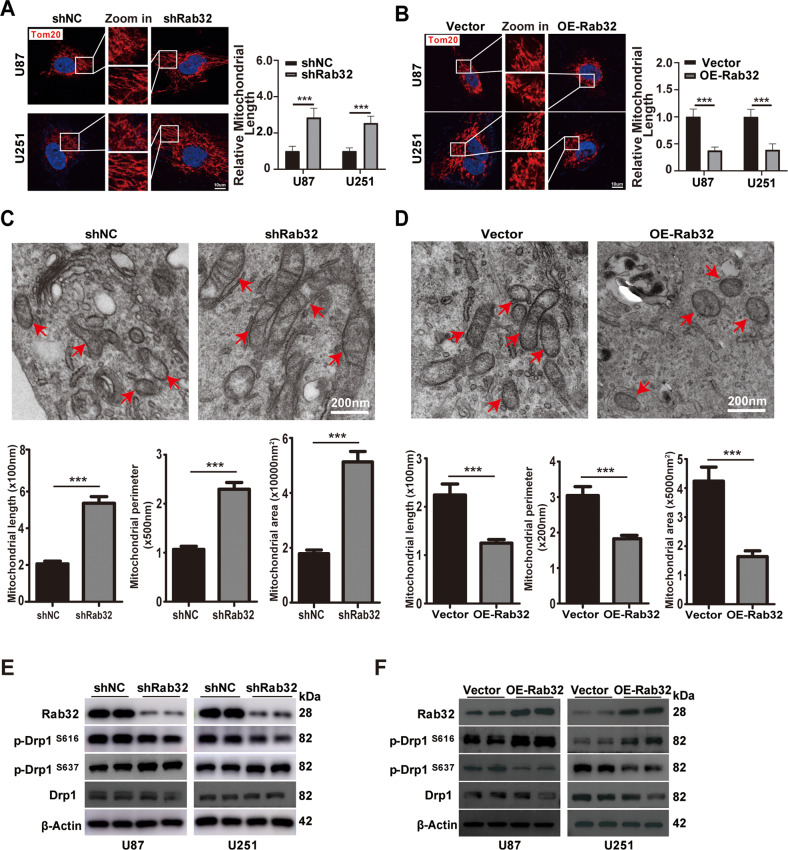
Rab32 regulates mitochondrial fission in glioma cells in a Drp1-dependent manner. **A**, **B** Representative immunofluorescence images of the mitochondrial network (Left panel) in treaded U87 and U251 cells. Red: Tom20, Bule: DAPI. Areas in the white squares are amplified and shown in the middle. The length of mitochondria was quantified by Image J (Right panel). *N* = 30 per group. Data shown are mean ± SD. Scale bar = 10 μm. **C**, **D** Transmission electron microscopy (TEM) photomicrographs of U87MG cells with Rab32 knockdown or overexpression (upper panel). Quantification of mitochondrial length, perimeter and area using ImageJ software (lower panel). Mitochondria are highlighted in red arrows. *N* = 30 per group. Data shown are mean ± SD. **E**, **F** Western blot analysis of the protein levels of phosphorylated Drp1 at Ser637 (p-Drp1^S637^) or Ser616 (p-Drp1^S616^) sites in indicated U87 and U251 cells. β-Actin was used as a loading control. ***P < 0.001 vs. NC groups.

Abnormal mitochondrial dynamics is served as a critical hallmark of GBM and leads to tumor cell migration, malignant progression, and therapy resistance [14, 25, 26]. We wondered whether Rab32 regulated mitochondrial dynamics in glioma that contribute to its role in GBM aggressiveness. The mitochondrial morphology of U87 and U251 cell lines was evaluated after Rab32 manipulations. The immunofluorescence images showed that knockdown of Rab32 by lenti-virus shifted mitochondrial dynamics towards fusion and led to increasing length of mitochondria (Fig. 3A). On the contrary, over-expression of Rab32 enhanced mitochondrial fission and resulted in short mitochondria pieces (Fig. 3B). We also examined the mitochondrial morphology of U87 cells upon Rab32 manipulations by Transmission Electron Microscopy (TEM). The results showed that the mitochondrial area, perimeter, and length were significantly increased in Rab32-silenced U87 cells. In contrast, over-expression of Rab32 exhibited the opposite effect (Fig. 3C, D).

Since Drp1 is a key regulator and reporter of mitochondrial fission, we then examined the expression levels of Drp1 after Rab32 manipulations. We found that silencing of Rab32 suppressed Drp1 phosphorylation at serine 616 (p-Drp1^S616^), which is involved in mitochondrial fission, and promoted Drp1 phosphorylation at serine 637 (p-Drp1^S637^), which mediated mitochondrial fusion (Fig. 3E and Fig. S4A). In contrast, Rab32 over-expression elevated the level of p-Drp1^S616^ and decreased the level of p-Drp1^S637^, with no dramatical changes in the total Drp1 level (Fig. 3F and Fig. S4B). We further examined the expression levels of two important mitochondrial fission-related factor, MFF and H-FIS1, in both U87 and U251 cells by western blot analysis. The protein levels of H-FIS1 or MFF showed no significant differences between the shRab32 and shNC groups. Similarly, no dramatic change was detected upon Rab32-overexpression (Fig. S4C–D). These results indicated Rab32 promoted Drp1-mediated mitochondrial fission in a MFF/H-FIS1-independent manner.

**Fig. 4:**
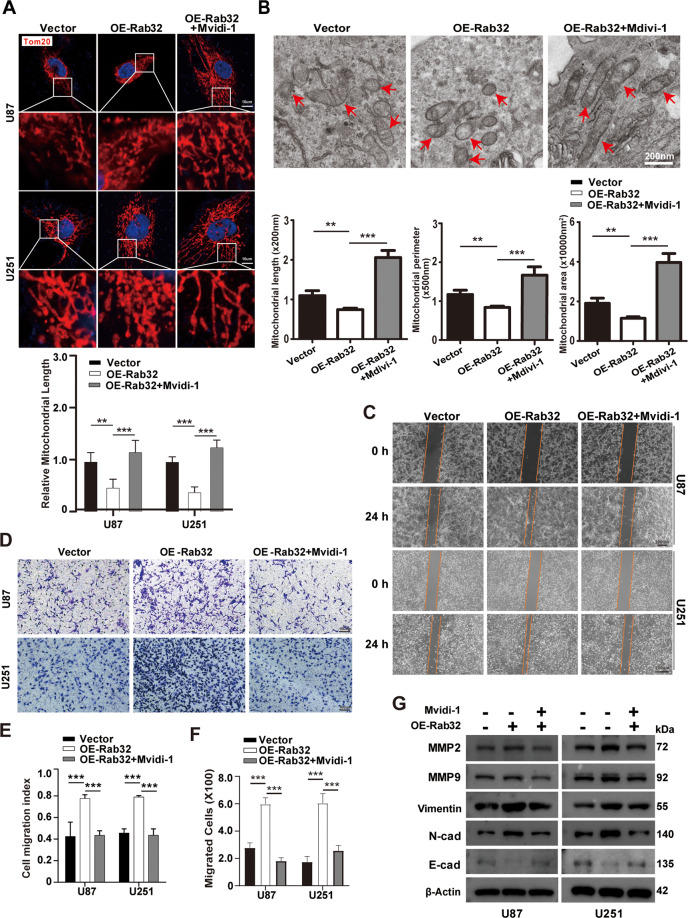
Drp1 inhibitor attenuates the effect of Rab32 overexpression on GBM malignancy. The U87 and U251 were transfected with OE-Rab32 plasmid and then treated with 50 μM Mdivi-1 for 24 h. **A** Representative immunofluorescence images of the mitochondrial network (upper panel) in treaded U87 and U251 cells. Red: Tom20, Bule: DAPI. Areas in the white squares are amplified and shown in the middle. The length of mitochondria was quantified by Image J (lower panel). Scale bar = 10 μm. *N* = 30 per group. Data shown are mean ± SD. **B** TEM photomicrographs of U87MG cells with Rab32 knockdown or overexpression (upper panel). Quantification of mitochondrial length, perimeter and area using ImageJ software (lower panel). Mitochondria are highlighted in red arrows. *N* = 30 per group. Data shown are mean ± SD. **C** The migratory ability of cells was analyzed using a wound-healing assay. Scale bar = 200 μm. **D** A transwell invasion assay evaluated the invasive capacity of cells. Representative images of cells crossing the membrane are shown. Scale bar = 100 μm. **E**, **F** Quantitative analysis of glioma cells’ migratory and invasive capacity in **C** and **D**, respectively. **G** Western blotting analysis was performed to detect the level of MMP2, MMP9, Vimentin, N-cadherin, and E-cadherin. β-Actin was used as a loading control. The data are shown as the mean ± SD of three replicates. **P < 0.01, ***P < 0.001 vs. NC groups.

We further treated the cells with Mdivi-1, a specific cell-permeable mitochondrial fission inhibitor that targeted Drp1, and found the increased mitochondrial fission caused by Rab32-overexpresssion was blocked, as was shown by immunofluorescence images (Fig. 4A). Similar results were found by Transmission Electron Microscopy that Mdivi-1 could partially reverse the reduction in mitochondrial area, circumference and length mediated by Rab32 overexpression in U87 cells (Fig. 4B). We further performed wound healing and cell invasion assays, and found Mdivi-1 blocked the increase of cell migration and invasion caused by Rab32-overexpression (Fig. 4C–F). In consistence, the elevated expressions of MMP2, MMP9, Vimentin, and N-Cadherin, as well as the reduced expression of E-Cadherin in Rab32-overexpression cells were restrained after Midiv-1 treatment (Fig. 4G and Fig. S5A). These data indicated that Rab32 promoted mesenchymal transition and GBM invasion through the regulation of Drp1-dependent mitochondrial fission.

### Rab32 transports Drp1 from cytoplasm to mitochondria

**Fig. 5:**
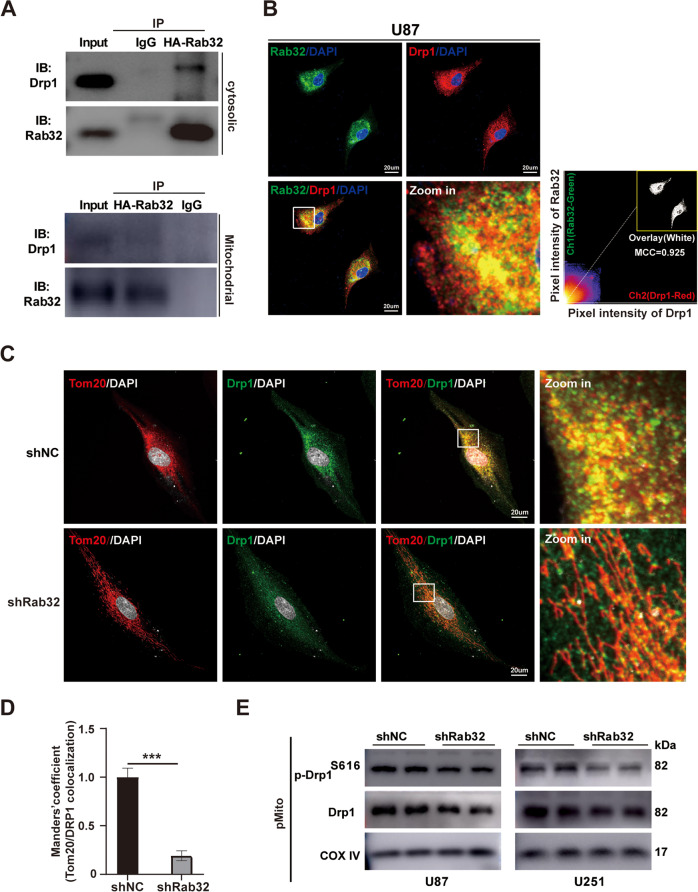
Rab32 transports Drp1 from the cytoplasm to the mitochondrial. **A** Immunoprecipitation assay evaluated the Rab32 interacted with Drp1 in the cytoplasm or on mitochondria. Rabbit IgG was used as a control. **B** Co-localization of Rab32 with Drp1 had been analyzed using immunofluorescence staining in treated U87 cells. The level co-localization was quantified by Manders Co-localization Coefficient (MCC) values using ImageJ software. Green: Rab32, Red: Drp1, Blue: DAPI. Scale bar = 20 μm. **C**, **D** Representative images and quantification of co-localization of Drp1 with mitochondria in treated U87 cells. Green: Drp1, Red: Tom20, White: DAPI. Scale bar = 20 μm. **E** Western blot analysis for protein expression of total Drp1 and p-Drp1^S616^ in mitochondrial fraction of U87 and U251 cells. COX IV was used as a control. The data are shown as the mean ± SD of three replicates. ***P < 0.001 vs. NC groups.

Like other Rab family proteins, Rab32 was reported to participate in intercellular transport [27, 28]. Here we wonder whether the transportation function of Rab32 was involved in mitochondrial fission of GBM. When we analyzed the protein-protein interaction network (PPI network) and looked for proteins that potentially interacted with Rab32 based on the STRING database (https://string-db.org/), we found that Drp1 associated closely with Rab32 (Fig. S5B). Our co-IP experiment with whole cell proteins and mitochondrial proteins also supported the direct interaction between Rab32 and Drp1 (Fig. 5A). We also co-stained Rab32 with Drp1, and found co-localization of these two proteins in the cytoplasm of GBM cells (Fig. 5B). Furthermore, we found that Drp1 was mainly located in the mitochondria, as was shown by the mitochondrial marker protein Tom20 (Fig. 5C). However, when we knockdown Rab32 in GBM cells, the localization of Drp1 in mitochondria was largely reduced (Fig. 5C, D). In parallel, when we isolated mitochondria from GBM cells and extracted proteins in this organelle, we found the expression levels of both p-Drp1^S616^ and total Drp1 were decreased upon Rab32 silence (Fig. 5E). Taken together, these data suggested that Rab32 promoted Drp1 transportation from cytoplasm to mitochondrial, then induced Drp1-mediated mitochondrial fission, leading to the migration and invasion of GBMs.

### Rab32-mediated mitochondrial fission is dependent on ERK_1/2_ activation

To further investigate the molecular mechanisms underlying Rab32-mediated mitochondrial fission, we analyzed the Rab32-related signal pathway by GO analysis based on RNA-sequencing data after Rab32 silencing (Fig. 2C). The data revealed that Rab32 depletion was associated with the regulation of ERK1 and ERK2 cascade, as well as negative regulation of MAPK cascade. Given that ERK signaling induced Drp1 phosphorylation at serine 616 and led to mitochondrial fission [29, 30], we wondered whether Rab32 regulates ERK signaling pathway.

**Fig. 6:**
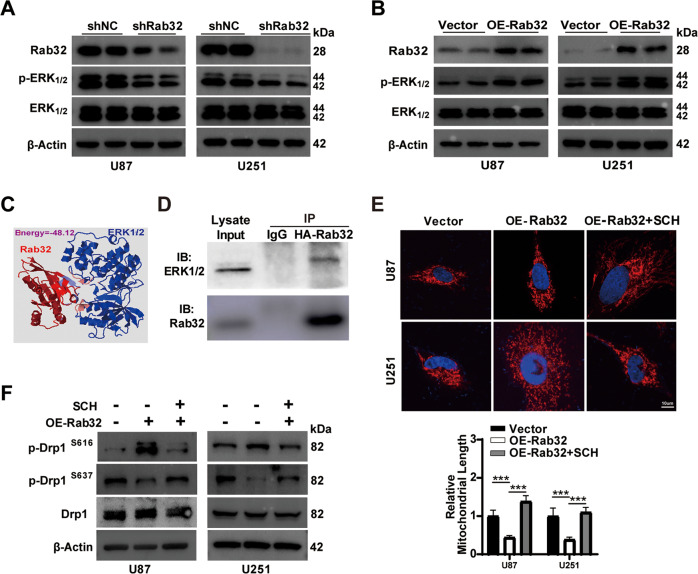
Rab32 recruited ERK for Drp1 activation. **A**, **B** Western blot analyzed the level of p-ERK_1/2_ in Rab32-silencing or overexpression U87 and U251 cells. β-Actin was used as a loading control. **C** Bioinformatics prediction of interaction between rab32 and ERK_1/2_ based on structure-based protein interaction interface. **D** Immunoprecipitation assay evaluated the Rab32 interaction with ERK_1/2_ in U87 cells. Rabbit IgG was used as a control. **E** Representative images and quantification of mitochondrial morphology assessed by Tom20 staining in OE-Rab32 U87 and U251 cells with or without ERK inhibitor SCH772984 (SCH, 1 μM) treatment for 24 h. **F** Western blot detected the levels of pDrp1 (Ser616 and Ser637) in OE-Rab32 U87 and U251 cells with or without SCH772984 treatment (SCH, 1 μM for 24 h). β-Actin was used as a control. The data are shown as the mean ± SD of three replicates. ***P < 0.001 vs. indicated groups.

Firstly, we found that silencing Rab32 reduced the level of phosphorylated ERK_1/2_ while the total ERK_1/2_ did not change dramatically in U87 and U251 cells (Fig. 6A and S6A). On the contrary, over-expression of Rab32 promoted phosphorylation and activation of EKR_1/2_ (Fig. 6B and S6B). Moreover, we applied the PRISM tool (http://cosbi.ku.edu.tr/prism) to predict their potential interaction interfaces, and our data showed that Rab32 interacted with ERK_1/2_ (Fig. 6C). We further performed Co-IP experiment and our result confirmed that Rab32 interacted with ERK_1/2_ (Fig. 6D). These data revealed that Rab32 interacted with ERK_1/2_ and activated ERK_1/2_ signaling pathway.

**Fig. 7:**
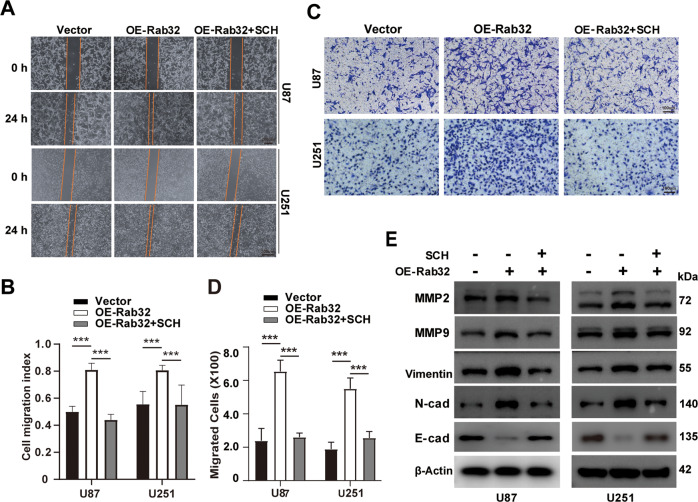
Rab32 regulated mesenchymal transition, migration, and invasion of GBM through modulating ERK activity. **A** Wound-healing assay was employed to analyze the migratory ability after OE Rab32- U87 and U251 cells stimulated with 1 μM SCH772984 for 24 h. Scale bar = 200 μm. **B** Quantitative analysis of the migratory capacity of glioma cells in **A**. **C** Transwell matrigel invasion assay was used to evaluate the invasive capacity of OE Rab32- U87 and U251 cells presence SCH772984 treatment. Scale bar = 100 μm. **D** Quantitative analysis of the invasion capacity of glioma cells in **C**. **E** Western blot analyzed the expression of MMP2, MMP9, Vimentin, N-cadherin, and E-cadherin after OE Rab32- U87 and U251 cells treated with 1 μM SCH772984 for 24 h. β-Actin was used as a loading control. Data are shown as the mean ± SD of three replicates; **P < 0.01, ***P < 0.001 vs. indicated groups.

Next, we treated GBM cells with SCH772984, an inhibitor of ERK_1/2_ signaling, and found it blocked Rab32-induced mitochondrial fission and suppressed Drp1 phosphorylation at Ser616 (Fig. 6E, F and S6C). To verify whether ERK inhibition affected Rab32-Drp1 interactions, we performed the Co-IP after Rab32-HA-transfected GBM cells treated with SCH772984 and the data showed that inhibition of ERK_1/2_ reduced the binding of Rab32 and Drp1, suggesting that the interaction of Rab32 with Drp1 is partly dependent on the ERK_1/2_ activation (Fig. S6D). Moreover, SCH772984 incubation of GBM cells also markedly abolished the enhancement of Rab32 on cell migration, invasion, and mesenchymal transition, as shown by wound healing assay, cell invasion assay, as well as protein analysis (Fig. 7A–E and S7A). These results together supported the notion that Rab32 promoted GBM migration, invasion, and mesenchymal transition via the regulation of ERK_1/2_-Drp1 signaling pathway.

### Downregulation of Rab32 inhibits GBM malignancy in vivo

**Fig. 8:**
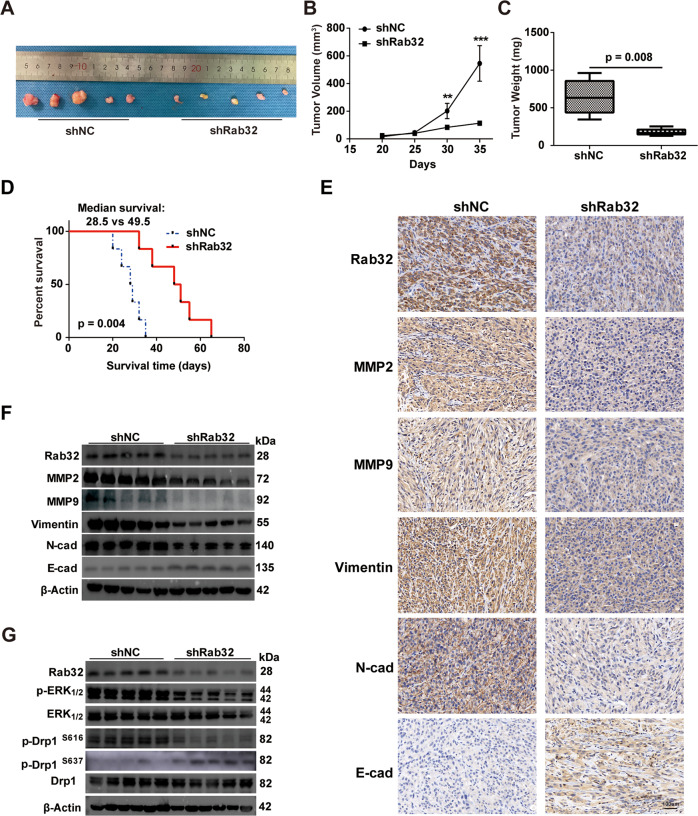
Downregulation of Rab32 inhibits GBM malignancy in vivo. **A** Representative images of tumors dissected from each group of mice were taken after nude mice were sacrificed. **B** The tumor volumes were measured and monitored every 5 days from each group of mice. NC or Rab32 silencing- U87 cells were subcutaneously injected into the flank of male BALB/c nude mice (*n* = 5 per group). **C** The weights of subcutaneous NC or Rab32 silencing- U87 cells tumors on the 35th day after injection. **D** Kaplan–Meier survival curve of intracranial in situ xenograft tumor nude mouse model with LV-shNC or LV-shRab32 transfected U87 cells (*n* = 6 per group). **E** Representative images of IHC staining of paraffin sections of in situ transplanted tumors in mice for Rab32, MMP2, MMP9, Vimentin N-cadherin, and E-cadherin. Scale bars = 100 μm. **F** Western blot analysis of Rab32, MMP2, MMP9, N-cadherin, E-cadherin, and Vimentin in situ xenograft tumors derived from NC- or Rab32 silenced -U87 cells. **G** Western blot analysis of Rab32, p-ERK_1/2_, ERK_1/2,_ p-Drp1^S616^, p-Drp1^S637^ and Drp1 in xenograft tumors derived from NC- or Rab32 silenced -U87 cells. β-Actin was used as loading control. All data are shown as the mean ± SD of three replicates. **P < 0.01, ***P < 0.001.

In order to evaluate the function of Rab32 on tumor formation in vivo, we further transplanted the GBM cells into the subcutaneous tissue of the nude mice. At 35 days post-injection, the tumor volumes of mice transplanted with Rab32-knockdown cells was significantly smaller than that with control cells (Fig. 8A, B). And the weight of tumors in the Rab32 knockdown group was less than that in control group, too (Fig. 8C). We also stereotaxically injected the GBM cells into the frontal cortex of the nude mice to assess tumor formation in the brain. Kaplan–Meier survival analysis showed that intracranial tumor-bearing mice with Rab32 knockdown had a tendency to prolong survival time compared to the control group, indicating the contribution of Rab32 on cancer malignancy (Fig. 8D). In addition, when we performed immunostaining on the intracranial tumor tissues, we found that knockdown of Rab32 reduced the expression of MMP2, MMP9, Vimentin, and N-Cadherin, while increased E-Cadherin protein level (Fig. 8E). Western blotting also supported these protein changes (Fig. 8F and Fig. S7B), which revealed the necessity of Rab32 in tumor invasion and mesenchymal transition. Finally, we confirmed that Rab32 knockdown suppressed p-ERK_1/2_ and p-Drp1^S616^ while increased p-Drp1^S637^ in the intracranial Xenograft mice (Fig. 8G and Fig. S7C). Taken together, our in vivo experimental results also demonstrated that Rab32 promoted mesenchymal transition and enhanced malignancy of GBM via the activation of ERK-Drp1 signaling pathway.

## Discussion

In the present study, we demonstrate the role of Rab32, as well as its downstream mechanisms in GBM. Our analysis on clinical samples showed that the expression level of Rab32 is high in glioma tissue, and positively correlated with tumor grade and poor prognosis. Both our cell culture and mouse xenograft studies revealed that Rab32 promoted the mesenchymal phenotype transition of GBM, as well as enhanced the migration and invasion of malignant cells. Moreover, we found that Rab32 transported Drp1 from the cytoplasm to the mitochondria, where ERK_1/2_ phosphorylated Drp1 at the site of Ser616, thereby induced mitochondrial fission. These findings suggested that Rab32 was severed as an oncogene and a novel therapeutic target for GBM.

Despite advances in diagnostic techniques and systemic treatment strategies, the prognosis of patients with GBM remains poor, mainly due to the high heterogeneity within the tumor and the characteristic infiltrative growth [31]. Several studies have identified that mesenchymal transition, as well as tumor migration and invasion are the key drivers of malignant progression and therapy resistance [32, 33]. However, the mechanisms underlying this process remain largely unclear. Therefore, elucidating the main regulators and signaling pathways of malignant glioma infiltration, especially the mesenchymal transition and tumor aggression, is expected to improve GBM therapeutic outcomes. The role of Rab32 in tumors is poorly explored, with only few studies reporting that Rab32 promotes tumor progression in hepatocellular and ovarian cancers [20, 34], but no study of Rab32 is performed on glioma. Here, we are the first to address the mesenchymal transition and cell invasion roles of Rab32 in GBM.

The Ras-related GTP binding protein (Rab) family consists of more than 60 human proteins and are thought to play an important role in regulating cellular signal transduction, vesicle transport, cytoskeleton dynamics, and membrane transport processes [35]. Previous studies have shown that some Rab proteins such as Rab1a, Rab21, and Rab26 are closely associated with the development and progression of many tumors, including glioma [36–38]. However, compared to other Rab proteins abundant in the cytoplasm, Rab32 is particularly concentrated in mitochondria and involved in mitochondrial dynamics [18]. Rybnicek et al. reported that Rab32 regulates the movement of mitochondria within differentiation of SH-SY5Y cells [39]. In this study, we revealed that the aggressive role of Rab32 in GBM was attributed to its regulation on mitochondrial fission.

But how did Rab32 regulate mitochondrial fission? Previous studies demonstrated that Rab32 modulates cellular physiological/pathological functions through dynamic intracellular transport of functional proteins [19, 40, 41]. Evidence from prior study suggested that the Rab32 subfamily proteins and their derivatives Rab29 and Rab38, could interact with GTPases such as Drp1, and modulate the regulation of mitochondrial dynamics [42]. Thinking that Rab32 is enriched in mitochondria, we hypothesized that Rab32 transported Drp1, the key regulator of mitochondrial fission, from cytoplasm to the mitochondria. In consistent with this proposal, we found Rab32 interacted with Drp1, and knockdown of Rab32 caused the diffusion of Drp1 from mitochondria to cytoplasm.

The mitochondrial fission process requires not only the attachment of Drp1 to the mitochondrial membrane but also the phosphorylation Drp1, which promotes Drp1 activity. Previous studies reported that Drp1 can be phosphorylated by either CDK1/5, ERK_1/2_, or PKA under certain conditions, which in turn affects mitochondrial morphology [43]. Our study here clarified that in GBM, Rab32 preferred to interact with ERK_1/2_ and activated its downstream signaling pathway. This finding not only provided insight into the mechanism underlying Rab32 regulation of mitochondrial fission, but also suggested a new potential therapeutic strategy for GBM patients by targeting the Rab32-ERK_1/2_-Drp1 axis.

Increasing evidence provides insight into mitochondrial dynamics, especially fission, which contributes to tumor metastasis [44]. Our study also revealed that Rab32 mediated mitochondrial fission and promoted migration and invasion of GBM cells. Further studies revealed that mitochondrial fission promoted the mobility, migration, and invasion of the tumor by regulation of metabolic reprogramming [15, 45, 46], Ca2 + -driven motility [47], lamellipodia formation [48], and ROS-mediated Rac1 activation and F-actin assembly [49]. Rab32 could promote lipid metabolic reprogramming efficiency [50], which supported migration, invasion, and metastasis of tumor cells [51]. Moreover, mitochondrial dynamics might regulate lipid metabolism [52]. Therefore, we speculated that Rab32 mediated mitochondrial fission, then regulated lipid metabolism, resulting in promoting migration, invasion, and metastasis of GBM.

In conclusion, our study provides evidence that Rab32 regulates ERK_1/2_/Drp1-dependent mitochondrial fission, resulting in mesenchymal transition, migration, and invasion capability of GBM. Given that regulation of mitochondrial dynamics has become one of the important strategies in current antitumor therapy, Rab32 may serve as a novel therapeutic target for GBM, especially for the mesenchymal subtype.

## Supplementary information

Original western blots used for CDD

Supplyment Figure ledengs

AUTHOR CONTRIBUTIONS

Supplyment Methords

ABBREVIIATION

aj-checklist

Fig. S1

Fig. S2

Fig. S3

Fig. S4

Fig. S5

Fig. S6

Fig. S7

## Data Availability

The data used to support the findings of this study are available from the corresponding author upon request.
