# Supplementary material for: Rab32 promotes glioblastoma migration and invasion via regulation of ERK/Drp1-mediated mitochondrial fission
Source: Cell Death Dis. 2023 Mar 15;14(3):198. doi: 10.1038/s41419-023-05721-3 (PMC10017813; doi:10.1038/s41419-023-05721-3)
Supplement: Supplementary file 2 — Supplyment Figure ledengs [file 41419_2023_5721_MOESM2_ESM.docx]

**Fig. S1** **A**. Differential mRNA expression of Rab32 in GBM and non-tumor samples according to Rembrandt datasets. **B and C.** The expression features of Rab32 in glioma of different clinicopathological grade in CGGA and Rembrandt datasets. **D and E**. The expression features of Rab32 in glioma of different molecular phenotype in CGGA and Rembrandt datasets. **F.** The expression features of Rab32 in glioma of different IDH-status in CGGA dataset. **G.** Kaplan-Meier analysis for correlation between Rab32 and survival of patients with gliomas in Rembrandt datasets.

**Fig. S2 A** and **B.** Quantitative analysis of the relative expression of MMP2, MMP9, Vimentin, N-cadherin, and E-cadherin protein using Image J software. β-Actin was used as a loading control. **C**. Western blot analysis of the expression of Rab32 after U87 and U251 cells were transfected with Rab32 overexpression (OE-Rab32) plasmid. β-actin was used as a loading control. **D**. A wound-healing assay was employed to analyze the migratory ability of OE-Rab32 U87 and U251 cells. Scale bar = 200 μm. **E**. The invasive capacity of OE-Rab32 U87 and U251 cells was analyzed using a transwell invasion assay. Representative images of cells crossing the membrane are shown. Scale bar = 100 μm. **F**. Western blot analysis for protein expression levels of MMP2, MMP9, Vimentin, N-cadherin, and E-cadherin in OE-Rab32 U87 and U251 cells. β-Actin was used as a loading control. **G** and **H.** Quantitative analysis of the relative expression of proteins in F using Image J software. The data are shown as the mean ± SD of three replicates; ***P < 0.001 vs. NC groups.

**Fig. S3 A.** The proliferation of U87 and U251 cells with Rab32 knockdown or overexpression was measured by colony formation. Colony numbers were counted under microscope. Data indicated as mean ± S.D (n ≥3 experiments). **B.** The rates of apoptosis in U87 and U251 cells with Rab32 knockdown or overexpression were determined by flow cytometric analyses using Annexin V-PE/7-AAD staining. The data are shown as the mean ± SD of three replicates; ns indicated no statistically significant difference, ***P < 0.001 vs. NC groups.

**Fig. S4 A and B.** Quantitative analysis of the relative expression of pDrp1(Ser637) and pDrp1(Ser616) protein after U87 and U251cells infected with Rab32 knockdown or overexpression. **C and D.** Western blotting analysis was performed to detect and quantify the level of MFF and FIS1 in U87 and U251 cells with Rab32 knockdown or overexpression. β-Actin was used as a loading control. The data are shown as the mean ± SD of three replicates; ns indicated no statistically significant difference, **P < 0.01, ***P < 0.001 vs. NC groups.

**Fig. S5 A.** Quantitative analysis of the relative expression of MMP2, MMP9, E-cadherin, N-cadherin, and Vimentin protein after OE-Rab32 U87 and U251 cells treated with 50 μM Mvidi-1 for 24 h. β-Actin was used as a loading control. The data are shown as the mean ± SD of three replicates; **P < 0.01, ***P < 0.001 vs. indicated groups. **B.** A protein-protein interaction network (PPI network) based on the STRING database was constructed to predict the potential interacting proteins (enrichment P < 1.0^-16^).

**Fig. S6 A and B**. Quantitative analysis of the relative expression of p-ERK_1/2_ and total ERK_1/2_ in U87 and U251 cells with Rab32 knockdown or overexpression. **C.** Quantitative analysis of the relative expression of pDrp1(Ser637), pDrp1(Ser616) after OE-Rab32 U87 and U251 cells treated with 1 μM SCH772984 for 24 h. β-Actin was used as a loading control. The data are shown as the mean ± SD of three replicates; **P < 0.01, ***P < 0.001 vs. indicated groups. **D.** Immunoprecipitation assay evaluated the Rab32 interaction with Drp1 in U87 cells treated with 1 μM SCH772984 for 24 h. Rabbit IgG was used as a control.

**Fig. S7 A.** Quantitative analysis of the relative expression of MMP2, MMP9, Vimentin, N-cadherin, and E-cadherin protein after OE-Rab32 U87 and U251 cells treated with 1 μM SCH772984 for 24 h. **B.** Quantitative analysis of the relative expression of MMP2, MMP9, Vimentin, N-cadherin, and E-cadherin protein in situ xenograft tumors. **C.** Quantitative analysis of the relative expression of Rab32, pDrp1(Ser637), and pDrp1(Ser616) protein in xenograft tumor tissues. β-Actin was used as a loading control. ns indicated no statistically significant difference. **P < 0.01, ***P < 0.001 vs. indicated groups.
