# Supplementary material for: Rab32 promotes glioblastoma migration and invasion via regulation of ERK/Drp1-mediated mitochondrial fission
Source: Cell Death Dis. 2023 Mar 15;14(3):198. doi: 10.1038/s41419-023-05721-3 (PMC10017813; doi:10.1038/s41419-023-05721-3)
Supplement: Supplementary file 3 — AUTHOR CONTRIBUTIONS [file 41419_2023_5721_MOESM3_ESM.docx]

**AUTHOR CONTRIBUTIONS**

Both corresponding authors (XZ and WH) and PC designed this project. PC, YL and BH conducted the experiments and processed data. PC, CY and SW draw the pictures and analyzed the data. YY, TL and ZL collected the clinical tissue samples. PC and BH wrote the manuscript. XZ, WH and TX reviewed and revised the manuscript. All authors have approved the final manuscript and have no conflicts of interest.
