# Supplementary material for: Rab32 promotes glioblastoma migration and invasion via regulation of ERK/Drp1-mediated mitochondrial fission
Source: Cell Death Dis. 2023 Mar 15;14(3):198. doi: 10.1038/s41419-023-05721-3 (PMC10017813; doi:10.1038/s41419-023-05721-3)
Supplement: Supplementary file 4 — Supplyment Methords [file 41419_2023_5721_MOESM4_ESM.docx]

**Cell apoptosis assay**

An apoptosis assay kit (Invitrogen, USA) was used to detect apoptosis in U87 or U-251 cells. Briefly, cells (5×10^5^/well) were harvested and then treated with 5 μL annexin V-FITC and 10 μL PI staining solution for 10 minutes in the dark at room temperature. Apoptosis was detected using a CytoFLEX flow cytometer and CytoFLEX analysis software (Beckman Coulter, USA).

**Cell proliferation and colony formation assays**

For the clonogenicity assay, every 10^3^ glioma cells were re-suspended in 1.5 mL of complete growth medium and then seeded into the wells of a 6-well plate. Medium was changed every three days. After 2 weeks of culture, cells were fixed in cold 4% paraformaldehyde and then stained with 1.0% crystalline violet solution. Colonies consisting of more than 50 cells were considered as a single colony. The visible colony numbers on each well were counted, and data were analyzed.

The proliferative potential of the cells was assessed using the CCK-8 assay. 10^3^ glioma cells treated with different concentrations of Mdivi-1 or SCH772984 was repeatedly inoculated into a 96-well plate and incubated for 24 h. Then, 10 mL of CCK-8 solution (Beyotime, Shanghai, China) was added to each well and incubated for 2 h. Optical density values at 450 nm were measured using an enzyme marker (BioTek, Winooski, VT, USA).

**Transmission electron microscope (TEM)**

First, Rab32-manipulated or drug-treated U87 glioma cells were fixed in 2.5% glutaraldehyde in 0.2 M HEPES overnight at 4°C. Cells were postfixed in 1% OsO4 for 60 min at room temperature, stained with 1% uranyl acetate, dehydrated in graded acetone solution and embedded in Poly 812 resin (90529-77-4, SPI). The area containing the cells was block mounted, sectioned at 70 nm and examined by electron microscopy (JOEL 1230, Japan).
