# Supplementary material for: Rab32 promotes glioblastoma migration and invasion via regulation of ERK/Drp1-mediated mitochondrial fission
Source: Cell Death Dis. 2023 Mar 15;14(3):198. doi: 10.1038/s41419-023-05721-3 (PMC10017813; doi:10.1038/s41419-023-05721-3)
Supplement: Supplementary file 5 — ABBREVIIATION [file 41419_2023_5721_MOESM5_ESM.docx]

**ABBREVIIATION**

GBM: glioblastoma multiforme;

Tom20: translocase of outer mitochondrial membranes 20 Kda;

MMP2: Matrix metalloproteinase 2;

MMP9: Matrix metalloproteinase 9;
