# Supplementary figures and images for: Rab32 promotes glioblastoma migration and invasion via regulation of ERK/Drp1-mediated mitochondrial fission
Source: Cell Death Dis. 2023 Mar 15;14(3):198. doi: 10.1038/s41419-023-05721-3 (PMC10017813; doi:10.1038/s41419-023-05721-3)

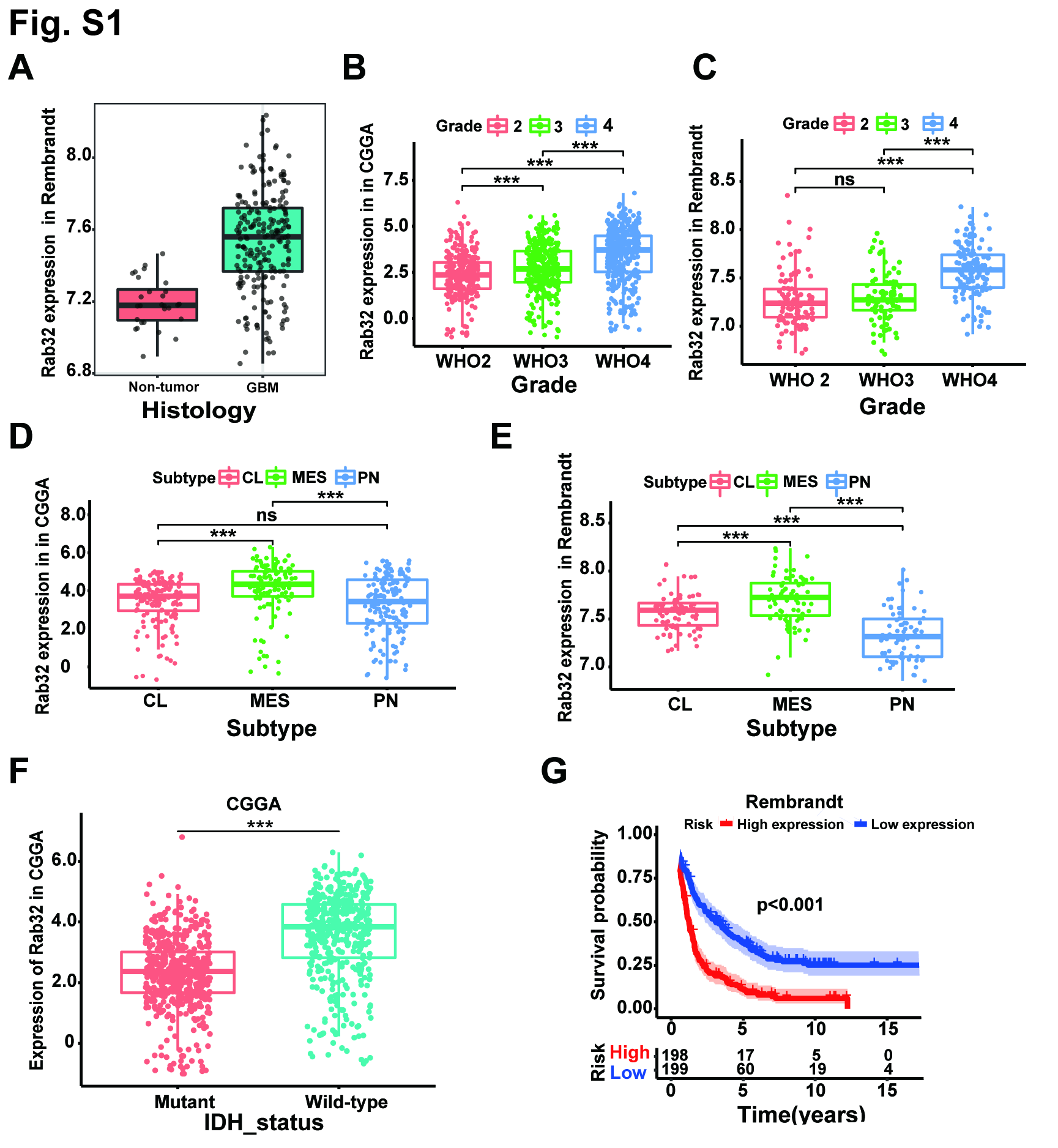

Supplement: Supplementary file 7 — Fig. S1 [file 41419_2023_5721_MOESM7_ESM.tif]

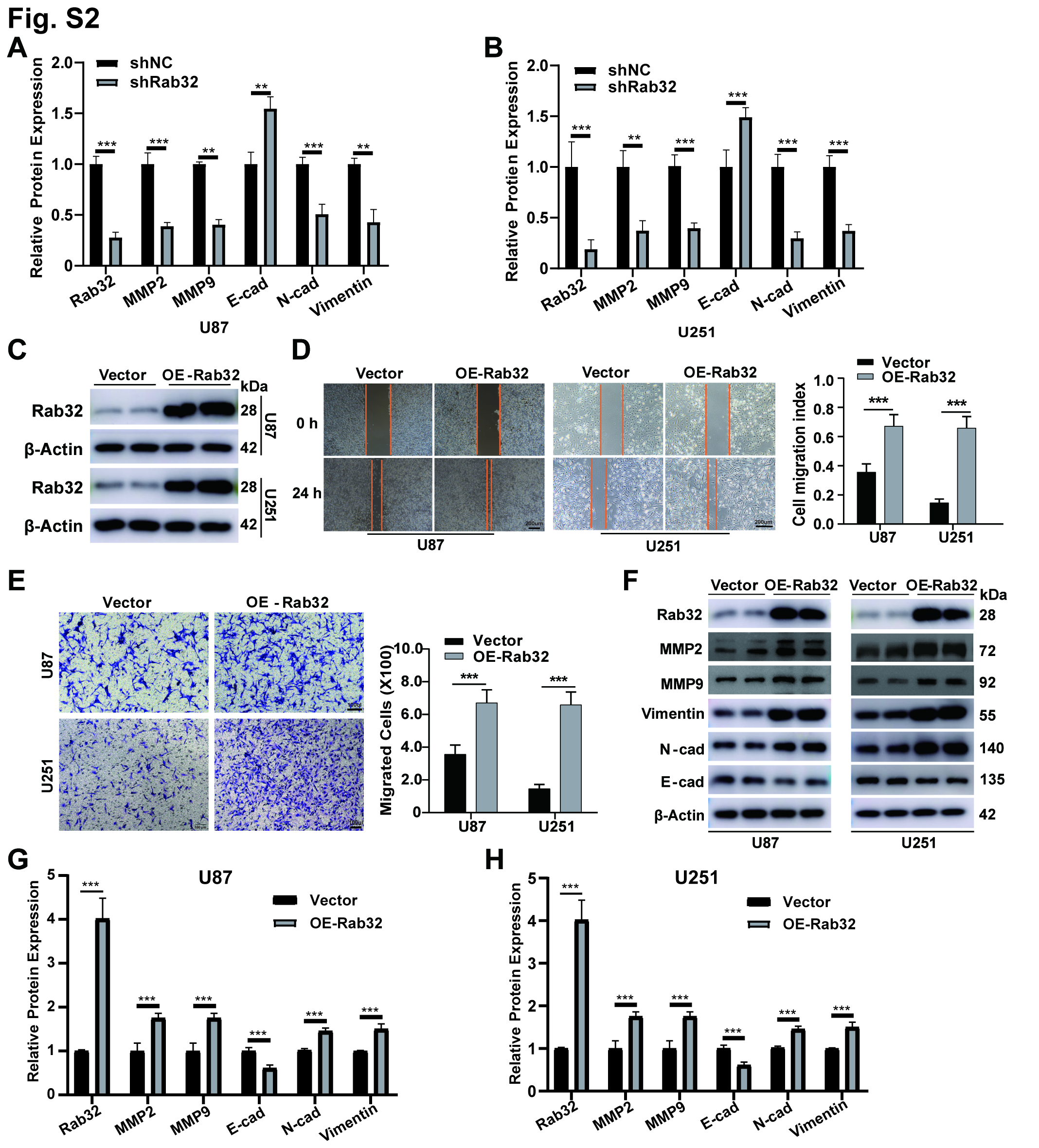

Supplement: Supplementary file 8 — Fig. S2 [file 41419_2023_5721_MOESM8_ESM.tif]

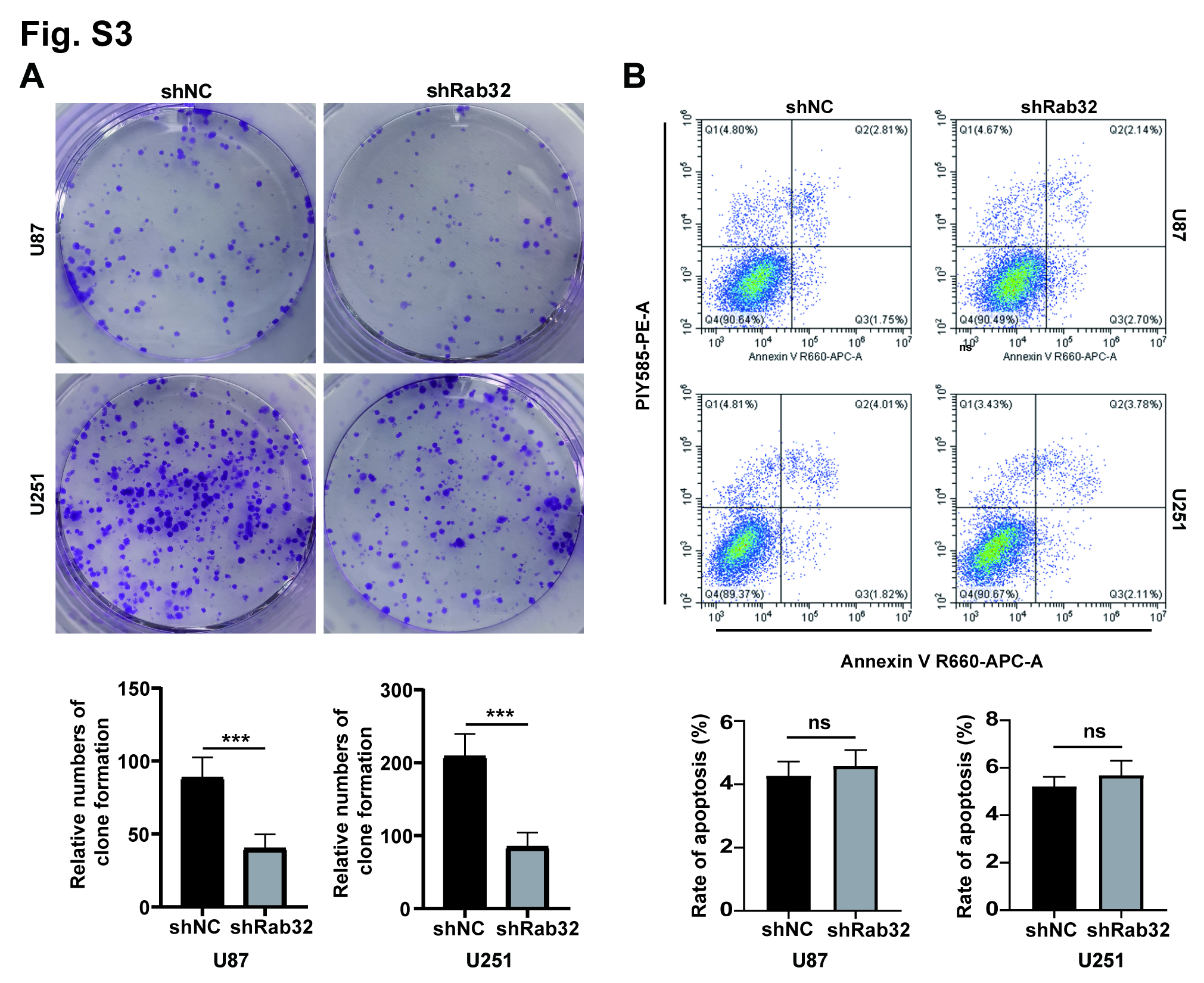

Supplement: Supplementary file 9 — Fig. S3 [file 41419_2023_5721_MOESM9_ESM.tif]

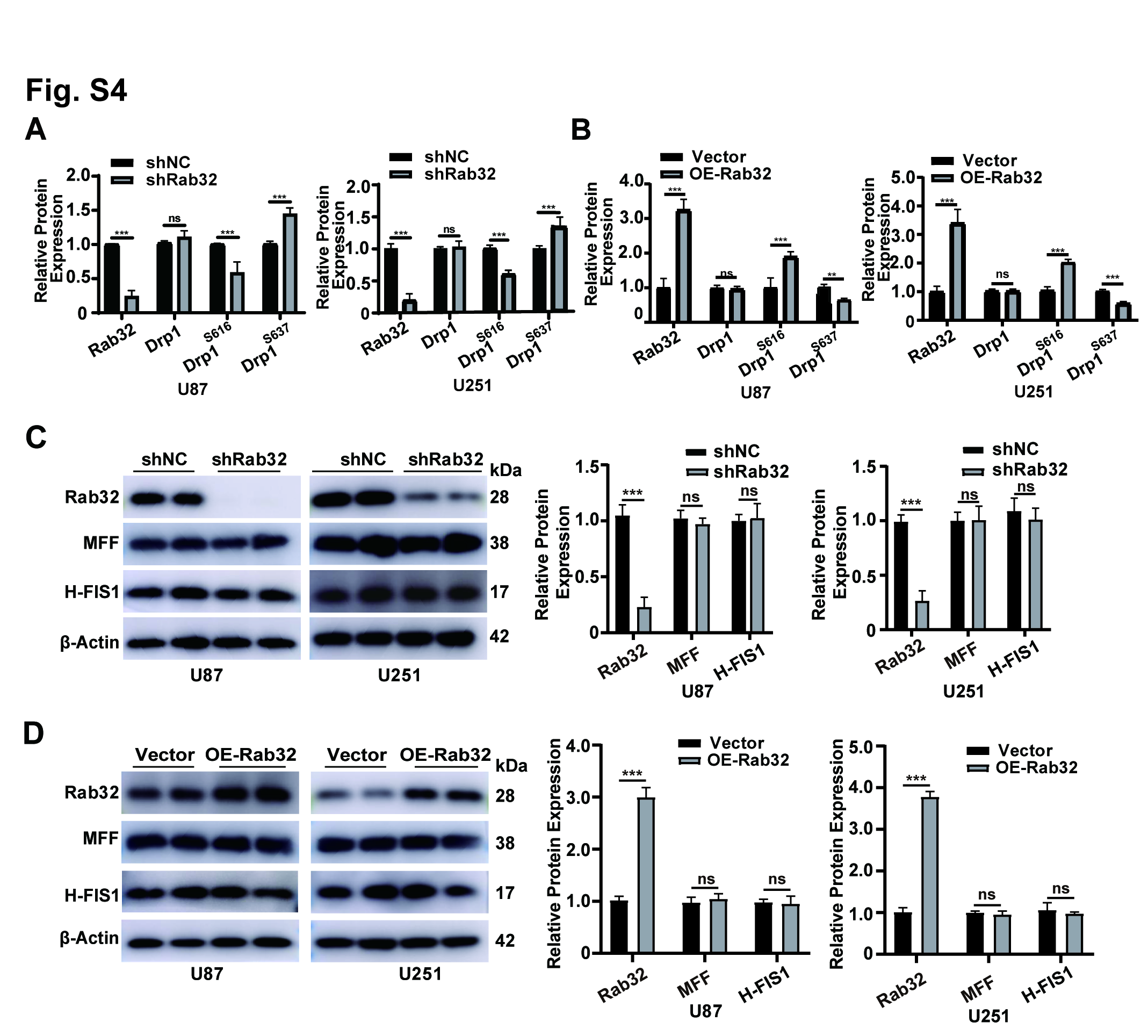

Supplement: Supplementary file 10 — Fig. S4 [file 41419_2023_5721_MOESM10_ESM.tif]

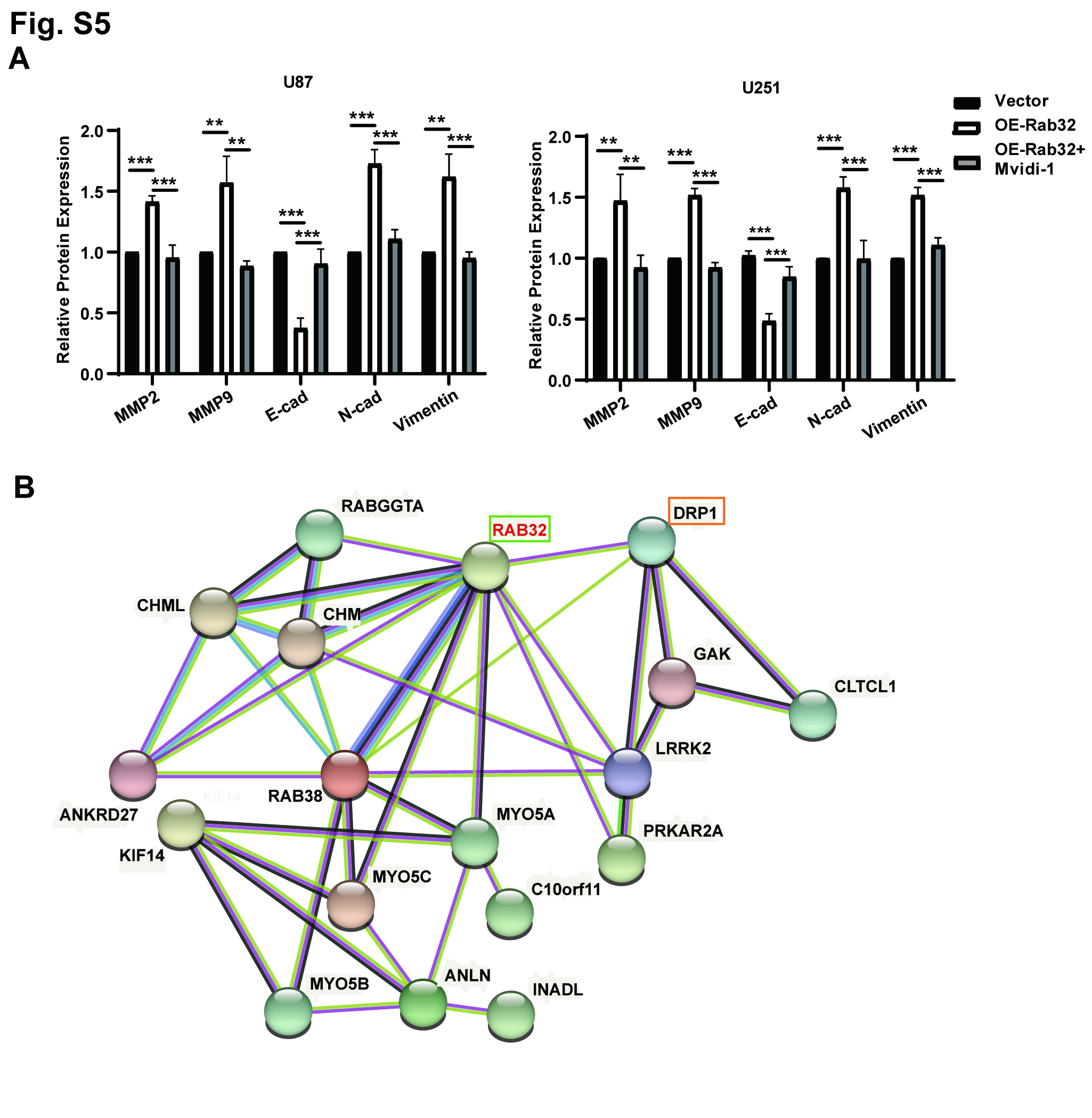

Supplement: Supplementary file 11 — Fig. S5 [file 41419_2023_5721_MOESM11_ESM.tif]

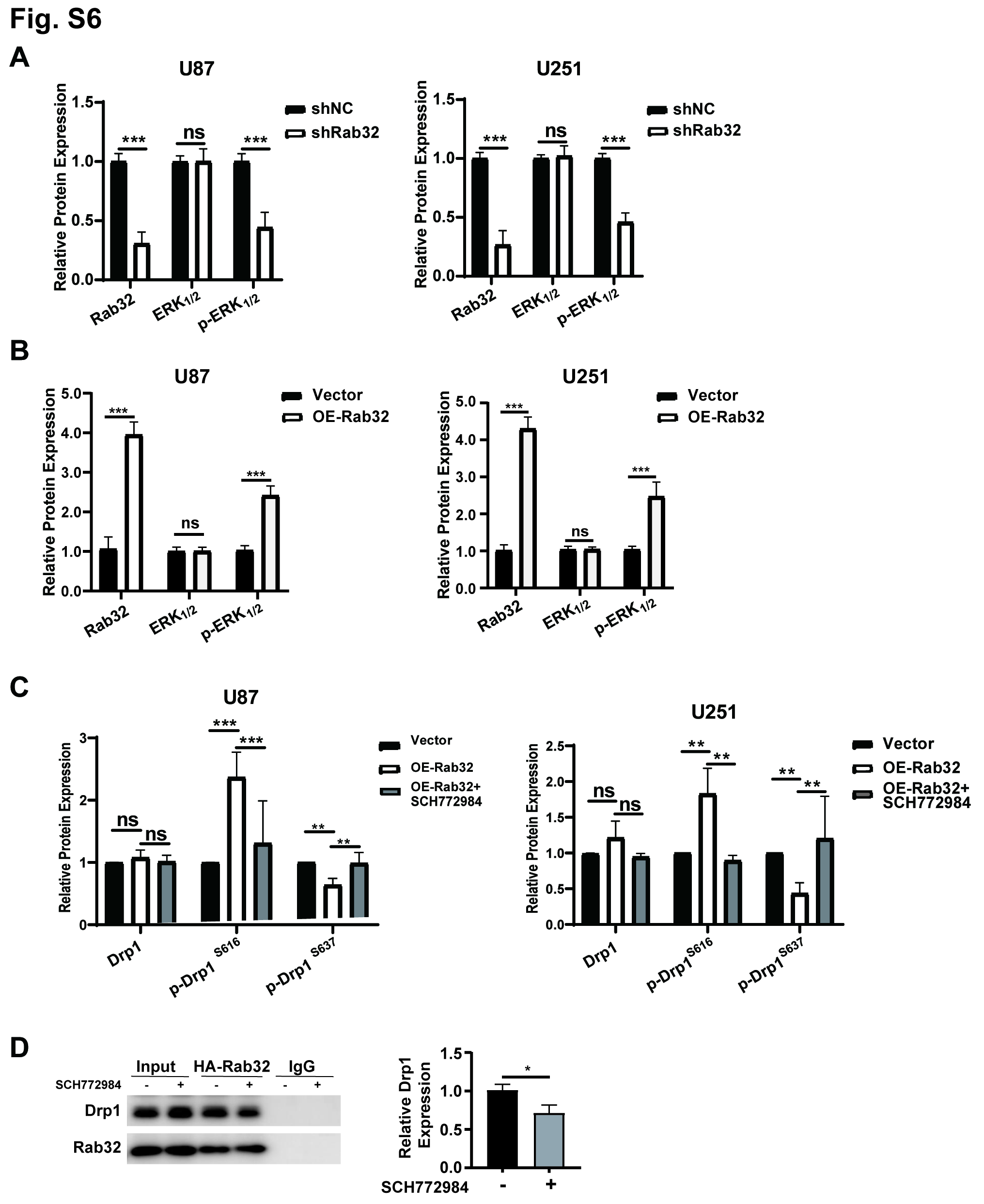

Supplement: Supplementary file 12 — Fig. S6 [file 41419_2023_5721_MOESM12_ESM.tif]

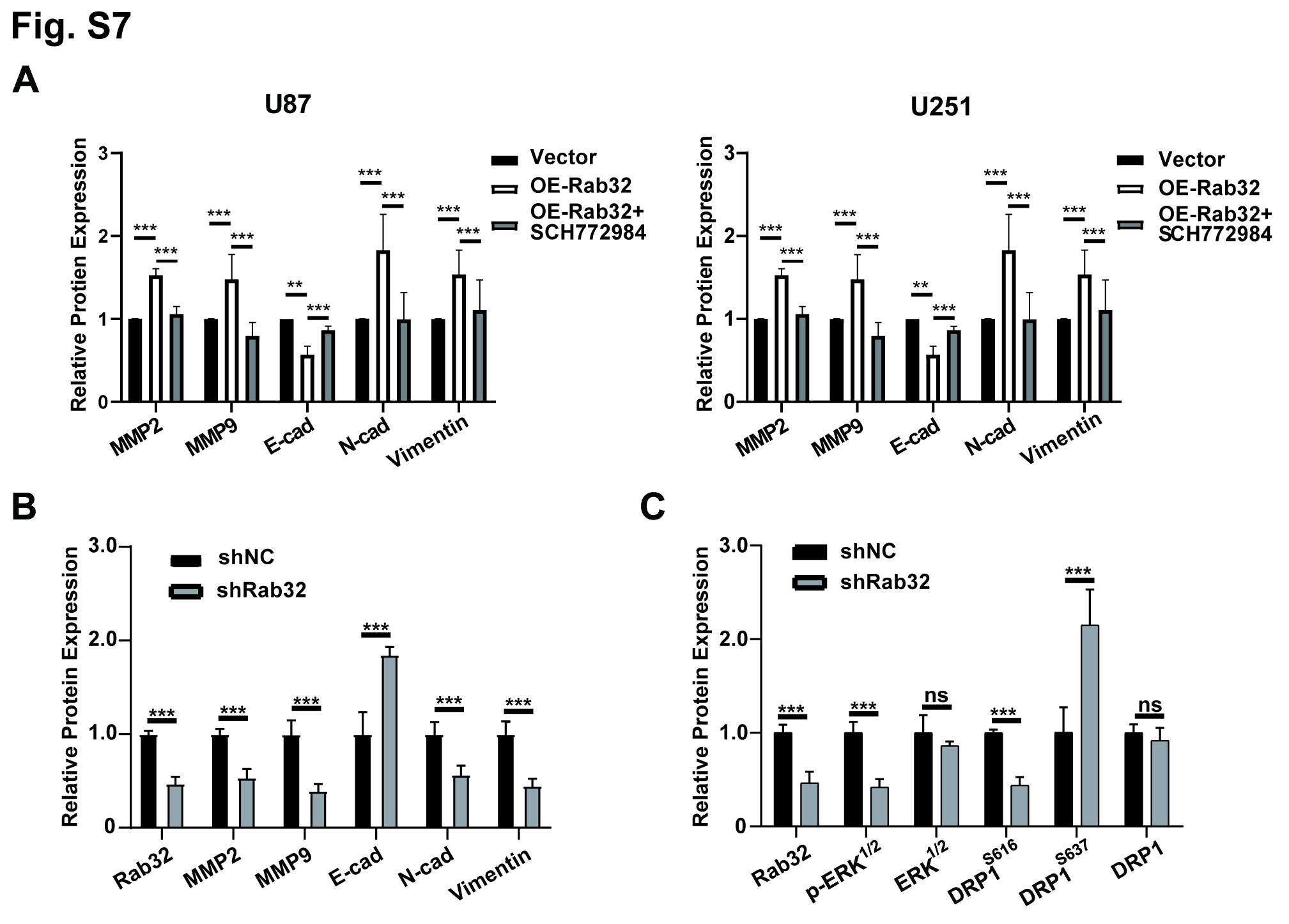

Supplement: Supplementary file 13 — Fig. S7 [file 41419_2023_5721_MOESM13_ESM.tif]
